# Change in alkaline phosphatase activity associated with intensive care unit and hospital length of stay in patients with septic acute kidney injury on continuous renal replacement therapy

**DOI:** 10.1186/s12882-018-1028-9

**Published:** 2018-09-20

**Authors:** Seung Don Baek, Jae-Young Kang, Hoon Yu, Seulgi Shin, Hyang-Sook Park, Mi-Soon Kim, Eun Kyoung Lee, So Mi Kim, Jai Won Chang

**Affiliations:** 1 Division of Nephrology, Department of Internal Medicine, Mediplex Sejong Hospital, Incheon, South Korea; 2 Division of Nephrology, Department of Internal Medicine, Sejong General Hospital, Bucheon, South Korea; 3 Division of Nephrology, Department of Internal Medicine, Gangneung Asan Hospital, University of Ulsan College of Medicine, Gangneung, Korea; 4 Department of Nursing, Asan Medical Center, University of Ulsan College of Medicine, Seoul, South Korea; 5 Division of Nephrology, Department of Internal Medicine, Dankook University College of Medicine, Cheonan-si, Chungnam South Korea; 6 Division of Nephrology, Department of Internal Medicine, Asan Medical Center, University of Ulsan College of Medicine, 388-1 Pungnap-dong Songpa-gu, Seoul, Republic of Korea

**Keywords:** Septic acute kidney injury, Alkaline phosphatase, Continuous renal replacement therapy

## Abstract

**Background:**

Evidence suggests that alkaline phosphatase attenuates inflammatory response in sepsis by lipopolysaccharide detoxification and adenosine triphosphate dephosphorylation. We sought to determine changes in alkaline phosphatase (AP) activity during septic acute kidney injury (AKI) and clinical parameters associated with AP activity.

**Methods:**

In this retrospective study, we investigated baseline (when initiating CRRT) and follow-up AP activity on day 3, and associated outcomes in patients who underwent continuous renal replacement therapy (CRRT) due to septic AKI.

**Results:**

We analyzed the baseline AP activity of 155 patients and day 3 AP activity in 123 patients. Baseline AP activity was not associated with renal or inflammatory biomarkers, or outcomes. It did not significantly differ between the 75 survivors and 80 non-survivors (*p* = 0.155). AP activity was higher on day 3 than at baseline (105 U/L [interquartile range, 79–156] vs 90 U/L [interquartile range, 59–133]). In particular, liver and bone isoforms increased significantly (*p* < 0.05), but intestine isoforms did not reach statistical significance (*p* = 0.367). In addition, day 3 AP activity showed a weak correlation with length of ICU stay (*r* = 0.213, *p* = 0.018) and length of hospital stay (*r* = 0.216, *p* = 0.017), but not with survival (*r* = − 0.035, *p* = 0.698).

**Conclusion:**

Endogenous AP activity significantly increased in patients with septic AKI. However, neither baseline nor follow-up AP activity was associated with survival.

## Background

Sepsis is the most common cause of death in critically ill patients and has high mortality rate despite recent advances [1]. As systemic inflammatory response evokes in sepsis, it leads to multiple organ dysfunction. Acute kidney injury (AKI) is one of the organs that most frequently fails, and it increases the risk for morbidity and mortality.

Several therapeutic interventions have been suggested to patients with sepsis from kidney injury [2]. Alkaline phosphatase (AP) is an especially promising enzyme that is under intense investigation. There are 4 different isoforms of AP including intestinal, liver-bone-kidney, placental, and the germ cell line [3]. The primary isoforms mostly originate from the liver and bone. Major defense mechanisms of AP include lipid A in lipopolysaccharide monophosphorylation and dephosphorylation of extracellular adenosine triphosphatase. AP has shown to have a protective role in not only sepsis [4, 5] but also inflammatory bowel disease [6] and cardiopulmonary bypass [7]. The kidney has been suggested to benefit from exogenous AP infusion in experimental [8] and preliminary clinical studies [9].

In this study, we measured total endogenous AP activity in patients with severe sepsis and kidney injury who required CRRT. We hypothesized that an increase in endogenous AP would be associated with beneficial outcomes in individuals at high risk for mortality. Baseline and follow-up AP activity with its isoforms were evaluated. We also determined the clinical outcomes associated with AP activity. Subsequently, we sought to find the clinical role of endogenous AP as a defense enzyme against septic kidney injury.

## Methods

### Patients

This is a retrospective observational study. We extracted data from all patients who underwent CRRT for septic AKI in ICU from January 1, 2014, to December 31, 2015, at the Asan medical center, a tertiary referral hospital. Septic AKI was defined as the simultaneous presence of the criteria for AKI [10] and the consensus criteria for sepsis [11]. We excluded patients with chronic dialysis, prior solid organ transplantation, stage IV malignancy, and patients younger than 18 years. Altogether, a total of 177 hospitalized patients were analyzed. Among those, AP activity was not available in 14 patients, because a blood sample was not drawn. Eight patients with sepsis of hepatobiliary origin were not included in the analysis to exclude direct liver injury as a mechanism of AP elevation. We measured AP activity when initiating CRRT and designated it as baseline value in our study. Follow-up AP activity on day 3 was not available in 32 patients who died within 3 days. Finally, the baseline AP activity of 155 patients and day 3 AP activity of 123 patients were analyzed.

Patients were followed up until hospital discharge for measurement of length of ICU stay, length of hospital stay, and mortality. ICU discharge was planned when a patient’s physiologic status was stabilized and the need for ICU monitoring and care was no longer necessary.

### Data collection and definitions

The study protocol was approved by the Ethics Committee of the Asan Medical Center. We reviewed medical records to investigate baseline characteristics and clinical outcomes. Demographics included age, sex, acute respiratory distress syndrome (ARDS) [12], mean arterial pressure, inotropic score, urine output, AKI stage [10], CRRT, mechanical ventilation, extracorporeal membrane oxygenation (ECMO), Sequential Organ Failure Assessment (SOFA) score, and laboratory values (i.e. white blood cells, hemoglobin, platelet, blood urea nitrogen, creatinine, NGAL, albumin, bilirubin, total CO_2_, CRP, procalcitonin, and baseline and day 3 AP activity). Plasma NGAL and AP activity were measured in patients who started CRRT during the study period. Plasma NGAL was measured using a particle-enhanced turbidimetric immunoassay. The upper limit of NGAL at our center was 1300 ng/mL. Plasma AP activity was measured using an alkaline phosphatase kit (SPIFE ALP-20; Helena Laboratories, USA) and automated electrophoresis system (SPIFE 3000; Helena Laboratories, USA) at the start of CRRT and 3 days after CRRT initiation for comparison. Inotropic score was calculated as follows [13]: (dopamine dose × 1) + (dobutamine dose × 1) + (adrenaline dose × 100) + (noradrenaline × 100) + (phenylephrine dose × 100). Urine output was presented as the mean value of 6-h collection immediately before CRRT initiation. Milliliter per kg per hour as urine output dimension was used in the analysis. All variables were measured at the start of CRRT.

Duration of mechanical ventilation was defined as the time from intubation to the time of final extubation or successful weaning off the ventilator. Spontaneous breathing during 48 h without a ventilator was considered successful weaning of mechanical ventilation. Duration of CRRT was determined as the time from CRRT start to successful discontinuation of CRRT which required at least 48 h free of CRRT, irrespective of transition to conventional hemodialysis.

### CRRT details

CRRT was initiated in patients with severe acidemia, uncontrolled hyperkalemia, and/or the presence of significant organ edema. CRRT was performed by commercially available pump-driven machines (Prisma or Prismaflex; Gambro) and ST 100 hemofilter with AN 69 membrane (Gambro). Dialysate and replacement fluid were hemosol B0 (Gambro) with the addition of potassium or bicarbonate if necessary. The default mode was continuous venovenous hemodiafiltration (CVVHDF) with a dialysate flow rate of 20 mL/kg/h, replacement flow rate of 20 mL/kg/h using the pre-dilution method, and blood flow rate of 150 mL/min. Nafamostat mesilate was used in selected patients with hypercoagulability.

### Statistical analysis

Patient characteristics were compared using descriptive statistics. Categorical variables were presented as numbers with percentage and continuous variables as mean with standard deviations or median with interquartile ranges. Differences between groups were assessed by Fisher’s exact test for categorical variables, and the Mann Whitney U or Wilcoxon signed rank test for continuous variables. Correlations between AP activity and variables were evaluated using the Spearman correlation coefficient which measured the statistical strength of the relationship. A 2-sided *p* value < 0.05 was considered statistically significant. We used SPSS version 14.00 (IBM Corp., Armonk, NY).

## Results

Patient characteristics are shown in Table 1. Mean age was 67.4 ± 13.6; 91 (58.7%) were men and 64 (41.3%), women. Mean SOFA score was 14.1 ± 2.6. The most common source of infection was of thoracic origin. During the study period, 80 (51.6%) patients died. The SOFA score and bilirubin concentration were higher in non-survivors than in survivors (*p* < 0.05). The platelet count, creatinine, and albumin concentration were lower in non- survivors (*p* < 0.05); serum NGAL level was not statistically different between the groups (*p* = 0.249).

**Table 1:** Characteristics of the 155 study patients

	Total (*n* = 155)	Survivors (*n* = 75)	Non-survivors (*n* = 80)	*P* value
Age	67.4 ± 13.6	65.9 ± 14.6	68.7 ± 12.5	0.199
ICU, n (%)				< 0.005
Medical	126 (81.3)	61 (81.3%)	65 (81.2%)	
Surgical	29 (18.7)	14 (18.7%)	15 (18.8%)	
CCI	4.24 ± 0.428	4.23 ± 0.421	4.25 ± 0.436	0.735
Moderate-to-severe liver disease^a^, n (%)	26 (16.8)	8 (10.7)	18 (22.5)	0.055
Male, n (%)	91 (58.7)	40 (53.3%)	51 (63.8%)	0.249
ARDS, n (%)	8 (5.2)	2 (2.7)	6 (7.5)	0.319
Mean arterial pressure (mmHg)	64.9 ± 14.4	65.7 ± 11.4	64.0 ± 16.7	0.460
Inotropic score	27.1 ± 21.6	25.6 ± 19.5	28.5 ± 23.4	0.392
Urine output, mL/kg/h	0.56 ± 0.87	0.7 ± 1.1	0.4 ± 0.5	0.015
AKI stage, n (%)				0.390
1	27 (17.4)	14 (18.7)	13 (16.2)	
2	56 (36.1)	23 (30.7)	33 (41.2)	
3	72 (46.5)	38 (50.7)	34 (42.5)	
Mechanical ventilation support, n (%)	138 (89.0)	64 (85.3)	74 (92.5)	0.242
ECMO support, n (%)	13 (8.4)	5 (6.7%)	8 (10.0)	0.647
SOFA score	14.1 ± 2.6	13.0 ± 2.3	15.2 ± 2.4	< 0.005
Infection source, n (%)				0.329
Thoracic	79 (51.0)	36 (48.0)	43 (53.8)	
Intra-abdominal	39 (25.2)	19 (25.3)	20 (25.0)	
Hematologic	10 (6.5)	6 (8.0)	4 (5.0)	
Urogenital	10 (6.5)	7 (9.3)	3 (3.8)	
Skin / soft tissue	8 (5.2)	5 (6.7)	3 (3.8)	
Unknown	9 (5.8)	2 (2.7)	7 (8.8)	
Laboratory value				
WBC (×10^3^/mm^3^)	13.8 ± 10.0	14.7 ± 10.3	13.0 ± 9.7	0.292
Hemoglobin (g/dL)	9.8 ± 2.1	9.8 ± 2.1	9.7 ± 2.0	0.883
Platelet	123.8 ± 95.7	147.5 ± 102.3	101.6 ± 83.7	< 0.005
BUN (mg/dL)	47.7 ± 25.5	45.0 ± 21.0	50.3 ± 29.0	0.198
Creatinine (mg/dL)	2.6 ± 1.5	2.9 ± 1.7	2.2 ± 1.2	0.007
NGAL (ng/mL)	920.6 ± 424.6	965.5 ± 405.7	880.2 ± 439.9	0.249
Albumin (g/dL)	2.1 ± 0.5	2.3 ± 0.5	2.0 ± 0.6	0.010
Bilirubin (mg/dL)	3.0 ± 5.2	2.0 ± 4.0	3.8 ± 6.0	0.029
Total CO_2_ (mmol/L)	16.0 ± 5.4	15.5 ± 5.5	16.5 ± 5.2	0.265
CRP (mg/dL)	14.6 ± 11.3	14.2 ± 9.1	14.9 ± 13.1	0.703
Procalcitonin (ng/mL)	51.9 ± 106.2	46.8 ± 100.2	56.8 ± 112.0	0.561

Data are expressed as n (%) or mean ± SD

*ARDS* acute respiratory distress syndrome, *CCI* Charlson Comorbidity Index, *CRP* C-reactive protein, *CRRT* continuous renal replacement therapy, *ECMO* extracorporeal membrane oxygenation, *NGAL* neutrophil gelatinase-associated lipocalin, *SOFA* Sequential Organ Failure Assessment, *WBC* white blood cells

^a^ Defined as cirrhosis with portal hypertension

**Fig. 1:**
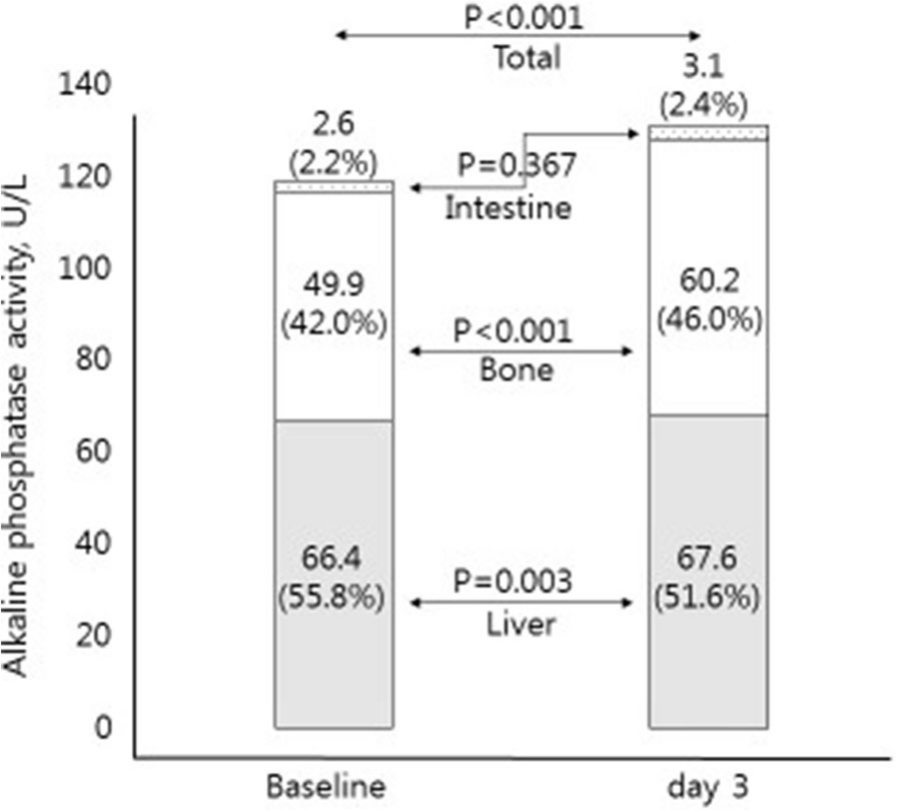
Change in alkaline phosphatase activity measured at baseline and on day 3. Data are expressed as mean values (%). The dotted box indicates intestine isoforms, the white box bone isoforms, and the gray box liver isoforms

Baseline and day 3 AP activity was 90 (range, 59–133) and 105 (range, 79–156), respectively. The mean difference was 19 (− 3 to 53), of which significantly increased compared with baseline (*p* < 0.001). We specifically measured each isoform at baseline and on day 3. Liver isoforms (55.8%) comprised the largest baseline AP activity, followed by bone (42.0%) and intestine (2.2%). Isoforms significantly increased on day 3 (*p* < 0.001), except for the intestine isoform (*p* < 0.367) (Fig. 1).

We analyzed AP activity according to survival to identify potential associations between AP activity and survival. However, there was no significant difference at baseline, day 3, and the mean difference of AP activity between survivors and non-survivors (Table 2).

**Table 2:** Total alkaline phosphatase activity and mean difference, measured at baseline and day 3

	All (*n* = 155)	Survivors (*n* = 75)	Non-survivors (*n* = 80)	*P* value
AP (U/L), baseline	90 (59–133)	79 (55–112)	92 (63–152)	0.155
AP (U/L), on day 3	105 (79–156)	105 (81–156)	103 (74–168)	0.697
Difference of AP activity (U/L)	19 (−3 to 53)	23 (−2 to 57)	16 (−5 to 45.8)	0.463

Data are expressed as median (interquartile range)

*AP* alkaline phosphatase

**Fig. 2:**
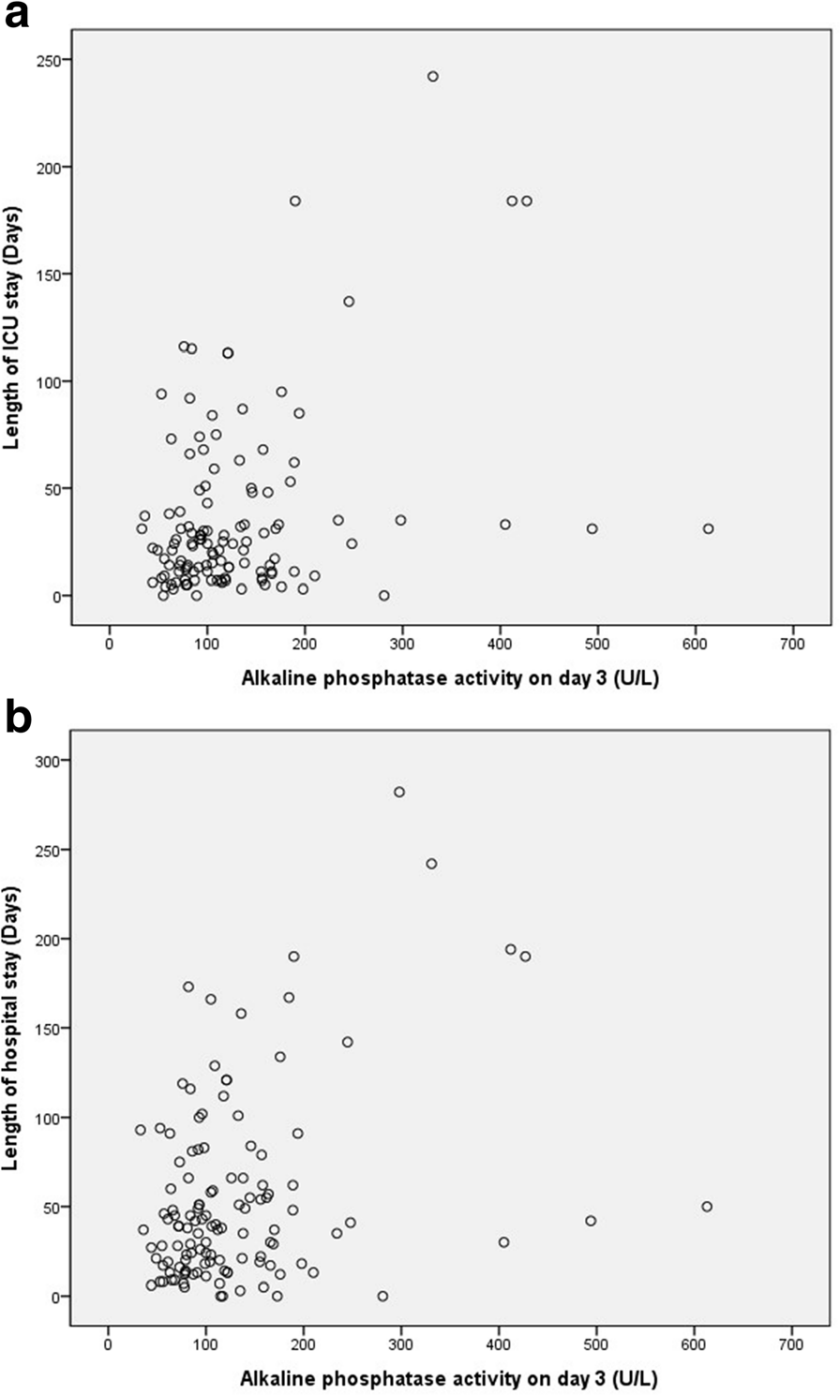
Scatter plot for alkaline phosphatase activity on day 3 versus length of ICU stay (**a**) and length of hospital stay (**b**)

Next, we performed a correlation analysis to find variables associated with AP activity. There were no correlated variables with baseline AP activity among baseline parameters and clinical outcomes (Tables 3 and 4). At the end of follow-up, mechanical ventilation duration, CRRT duration, length of ICU stay, and length of hospital stay were 9 (3–23), 7 (4–17), 17 (7–33), and 36 (13–62), respectively. Among these, length of ICU and hospital stay showed a weak correlation with day 3 AP activity (*r* = 0.213, *p* = 0.018, and *r* = 0.216, *p* = 0.017, respectively, Table 5 and Fig. 2). The mean difference of AP activity was not associated with length of ICU or hospital stay (*r* = − 0.046, *p* = 0.618, and *r* = − 0.034, *p* = 0.714, respectively).

**Table 3:** Correlation of alkaline phosphatase activity at baseline and urinary and inflammatory biomarkers

	Creatinine	Urine output	NGAL	Procalcitonin	CRP
AP (U/L), baseline	Correlation coefficients (*p* value)				
All (*n* = 155)	0.101 (0.212)	−0.096 (0.236)	0.091 (0.296)	−0.026 (0.744)	0.015 (0.851)

*AP* alkaline phosphatase

**Table 4:** Correlation of alkaline phosphatase activity at baseline and clinical outcomes

	MV duration	CRRT duration	Length of ICU stay	Length of hospital stay	Survival
AP (U/L), baseline	Correlation coefficients (*p* value)				
All (*n* = 155)	0.094 (0.245)	0.075 (0.351)	0.094 (0.247)	0.100 (0.218)	0.115 (0.156)

*AP* alkaline phosphatase, *CRRT* continuous renal replacement therapy, *MV* mechanical ventilation

**Table 5:** Correlation of alkaline phosphatase activity measured after 72 h, mean difference and clinical outcomes

	MV duration	CRRT duration	Length of ICU stay	Length of Hospital stay	Survival
AP (U/L), day 3	Correlation coefficients (*p* value)				
All (*n* = 56)	0.156 (0.086)	0.126 (0.165)	0.213 (0.018)	0.216 (0.017)	−0.035 (0.698)
AP (U/L), mean difference	Correlation coefficients (*p* value)				
All (*n* = 56)	−0.073 (0.426)	−0.045 (0.621)	− 0.047 (0.606)	−0.030 (0.746)	− 0.067 (0.465)

*AP* alkaline phosphatase, *CRRT* continuous renal replacement therapy, *MV* mechanical ventilation

Finally, neither day 3 nor the mean difference of AP activity was associated with survival (*r* = − 0.035, *p* = 0.698, and *r* = − 0.067, *p* = 0.465, respectively).

## Discussion

We found that total AP activity modestly increased in patients following septic AKI, and mostly consisted of liver and bone isoforms. Follow-up AP activity on day 3 showed a weak, but positive correlation with length of ICU and hospital stay. However, neither baseline nor follow-up AP activity increase was associated with survival.

To our knowledge, changes in endogenous AP activity among patients with septic AKI has not been demonstrated in the literature. In an animal study, an immediate decline in serum AP activity following the administration of LPS was reported [14]. The AP dephosphorylating enzyme has been shown to be depleted following an ischemic insult, especially in the kidney [15]. In addition, one study reported that AP activity decreased after cardiothoracic surgery in infants [16]. Increased AP turnover might explain the attenuation of the enzyme activity. Unlike data reported in patients undergoing cardiopulmonary bypass, we found that total AP activity after 3 days modestly increased in patients with sepsis and AKI. As LPS initiates inflammatory reaction and organ damage in sepsis, it would activate host defense mechanism to promote AP activity. Specifically, liver and bone isoforms had the highest increase. Total AP activity at baseline in our study was similar to the normal range (20–140 U/L) [17]. In the literature, 9074 critically ill patients showed baseline AP range of 57–93 U/L and maximal of 54–108 U/L [18], which was comparable to our study. We believe that the reason only liver and bone isoforms increased is that the liver and bone isoforms are predominantly involved in inflammation [3]. However, it might simply reflect statistical limitations due to our small sample size. A large cohort study is warranted to determine the real difference in AP increase between each isoform.

In our study, we believe that the increase in AP activity was a host response to septic injury. First, AP removal during hemodiafiltration is not feasible, as AP is a large molecule (> 65,000 Da) [16] and the Sieving coefficient of the molecule through a membrane is near zero. Even considering the dilutional effect, the increase is significant. Second, we did not include patients with hepatobiliary infections in the analysis to avoid the possibility that AP increased from direct liver injury. Third, AP activity has been reported to be depleted during ischemia [15]. The increase in AP in our study is a novel finding. Taken together, the increase in AP might be an endogenous reaction to modulate the inflammatory cascade in patients with septic AKI.

Despite numerous clinical trials to improve clinical outcomes of patients with sepsis, mortality is still substantial. AP has been highlighted to be a component of the host defense against inflammation. Exogenous AP infusion treatment has shown a potential role as one of the therapeutic interventions in patients with septic shock. Amongst LPS in gram-negative bacteria, lipid A toxic moiety has been shown to cause the inflammatory cascade. Dephosphorylation of LPS renders monophosphoryl lipid A virtually non-toxic and shows an antagonistic effect by competing with LPS in host immunity [19]. In a small controlled study, AP treatment showed improved outcomes by protecting renal function [5]. In this study, however, we could not identify an association between endogenous AP activity and patient survival. When analyzing the association of AP difference with survival, no association was seen.

In a previous study, lower AP activity post-operatively was associated with increased procalcitonin level in infants after cardiothoracic surgery [16]. In our study, we could not identify an association between baseline AP activity and inflammatory markers such as CRP and procalcitonin. We demonstrated that follow-up AP activity, although around a normal range, was weakly correlated with length of ICU and hospital stay. There was also a trend toward elevated follow-up AP activity and increased mechanical ventilation duration. The mechanism in patients with sepsis appears to be different from that of patients undergoing cardiovascular surgery whose AP activity has been reported to decrease [20]. Our present study had limitations that prevented from discussing the exact pathophysiology. Further studies are warranted to evaluate factors affecting changes in AP activity.

Based on our results, elevated follow-up AP activity along with the prolonged length of ICU and hospital stay suggested a promise of exogenous AP treatment to decrease the morbidity of patients. Considering the high mortality of patients with sepsis, the amount of endogenous AP might not be enough to counteract systemic inflammatory response and multi-organ dysfunction without exogenous supplement. There might be a beneficial role of exogenous AP infusion in facilitating patient recovery. Renal parameters were improved in septic AKI with calf intestinal AP in a prospective randomized study [21]. Combinations with other cytokine antagonizing mediators might achieve an optimal approach to the management of patients with sepsis. Based on our study, however, the performance of endogenous AP activity as a predictor of outcomes was poor. Future research could elucidate a definitive role of exogenous AP treatment, as it is beyond the scope of the present study to address the benefits of AP treatment.

We acknowledge several limitations. First, we studied a predefined population of interest and did not include all cases with sepsis. We confined the study population to patients at high risk for mortality who were diagnosed with septic AKI and supported by CRRT. Without appropriate control patients, it might lead to selection bias. However, we believe that it would be of interest to study the endogenous AP reaction and associated outcomes in patients with severe sepsis and kidney failure with significant renal inflammation and hypoxia because AP enzymes have been shown to predominantly protect the kidneys [22]. Second, our study has limited generalizability due to its small sample size, which originated from a single center. The study’s retrospective design also warrants caution in the interpretation of a causal relationship. Third, we collected AP activity only twice, which had limited information regarding the longitudinal changes in AP activity during the evolution of sepsis.

## Conclusion

Total AP activity modestly increased in the patients with septic AKI who required CRRT. Elevated follow-up AP activity was associated with a longer length of ICU and hospital stay. However, there was no association between endogenous AP activity and survival in patients with septic AKI, limiting its role as a prognostic indicator of mortality. Our study demonstrated that endogenous human AP activity during severe sepsis showed limited reserve against septic insult.

## Abbreviations

AKI: Acute kidney injury
AP: Alkaline phosphatase
ARDS: Acute respiratory distress syndrome
CCI: Charlson Comorbidity Index
CI: Confidence interval
CRRT: Continuous renal replacement therapy
ECMO: Extracorporeal membrane oxygenation
ICU: Intensive care unit
MV: Mechanical ventilation
NGAL: Neutrophil gelatinase-associated lipocalin
OR: Odds ratio
SOFA: Sequential Organ Failure Assessment
